# Prevalence of Different Combinations of Antiepileptic Drugs and CNS Drugs in Elderly Home Care Service and Nursing Home Patients in Norway

**DOI:** 10.1155/2016/5153093

**Published:** 2016-07-20

**Authors:** Kjell H. Halvorsen, Cecilie Johannessen Landmark, Anne Gerd Granas

**Affiliations:** ^1^Department of Pharmacy, Faculty of Health Sciences, The Arctic University of Norway, Tromsø, Norway; ^2^Department of Life Sciences and Health, Faculty of Health Sciences, Oslo and Akershus University College of Applied Sciences, Oslo, Norway; ^3^The National Center for Epilepsy, Oslo University Hospital, Oslo, Norway

## Abstract

*Introduction*. Antiepileptic drugs (AEDs) are used to treat different conditions in elderly patients and are among the drug classes most susceptible to be involved in drug-drug interactions (DDI). The aim of the study was to describe and compare use of AEDs between home care service and nursing home patients, as these patients are not included in nationwide databases of drug utilization. In the combined population, we investigate DDI of AEDs with other central nervous system- (CNS-) active drugs and DDIs involving AEDs in general.* Materials and Methods*. Point-prevalence study of Norwegian patients in home care services and nursing homes in 2009. At the patient level, we screened for different DDIs involving AEDs.* Results*. In total, 882 patients (7.8%) of 11,254 patients used AEDs and number of users did not differ between home care services and nursing homes (8.2% versus 7.7%). In the combined population, we identified 436 potential DDIs in 45% of the patients.* Conclusions*. In a large population of elderly, home care service and nursing home patients do not differ with respect to exposure of AEDs but use more AEDs as compared to the general population of similar age. The risk of DDIs with AEDs and other CNS-active drugs should be taken into consideration and individual clinical evaluations are assessed in this population.

## 1. Introduction

Due to their complex pharmacokinetic properties, antiepileptic drugs (AEDs) are among the drug classes most susceptible to be involved in drug-drug interactions (DDIs). In addition, the risk of pharmacodynamic interactions is augmented by concomitant prescribing with other central nervous system- (CNS-) active drugs [1]. The use of AEDs has increased during the last decade [2–4] because, in addition to treating epilepsy, AEDs are used to treat numerous other diseases and symptoms such as neuropathic pain (e.g., gabapentin and pregabalin), bipolar disease (e.g., lamotrigine), and migraine (e.g., valproate and topiramate) [2, 5–7].

Ageing in itself causes a reduction in hepatic and renal function, which subsequently altered blood flow, drug-protein binding, volume of distribution, clearance, and elimination half-life [8]. Pharmacokinetic considerations are important in this age group [9], particularly since many users of AEDs are older patients ≥65 years with a range of comorbidities and polypharmacy. This increases the risk for unintended pharmacokinetic and dynamic DDIs in this age group.

During the past 15 years, automatic drug dispensing or multidose drug dispensing, where patients receive medicines in small, prepackaged, and labelled plastic bags, has become a major dispensing method in Norway. The error rate of this automatic system is highly favourable compared to manual dispensing resulting in increased patient safety. Still, the frequencies of drug-related problems, that is, pharmacokinetic DDIs, are of concern [10].

Few studies have investigated DDIs involving AEDs in specialized health care settings, such as home care services and nursing homes. With more than 160,000 patients receiving home care services and about 43,000 patients in nursing homes, these settings constitute the largest health care service level in Norway. Patients receiving home care services are visited by a health care professional, that is, nurse, up to several times a day depending on caring needs [11]. Cognitive impairment affects about 30% of the patients. Patients in nursing homes are considered even more frail as most of them suffer from multiple chronic comorbid conditions, and 80% of these patients have cognitive impairment [12]. To improve the quality of drug therapy for older patients in these settings, it is important to conduct pharmacoepidemiological or drug utilization studies focusing on the identification of potentially inappropriate medications and potential DDIs.

The aim of the study was to describe and compare use of AEDs between home care service and nursing home patients, as these patients are not included in nationwide databases of drug utilization. Furthermore, in the combined population, we investigate potential DDIs involving AEDs and other central nervous system- (CNS-) active drugs and DDIs involving AEDs in general. This is an important concern for prescribers and clinical pharmacists when reviewing the appropriateness of drug therapy in the elderly.

## 2. Materials and Methods

### 2.1. Study Population

In this point-prevalence study, we received information about patients' drug use from Farmaka AS (Alliance Health), one of three suppliers of multidose dispensed drugs in Norway, on September 9th 2009. The company market share was ca. thirty percent of all multidose dispensed drug patients in home care services and nursing homes. The patients lived in all parts of Norway, except the less populated northern region. This is a unique source with regard to details of the prescribed drug, strength, dosage, formulation, and patient demographics, that is, not extractable from the Norwegian prescription database (NorPD) [13]. Furthermore, the NorPD does not reveal whether a patient receives home care services or not or any drug information about nursing home patients.

We included only patients aged 65 years and older and using ≥1 drug and those who received home care services or lived in nursing homes. For each patient, the following variables were provided: age, gender, setting (home care service or nursing home), and all regular multidose dispensed drugs (drug name, strength, formulation, and dosage). Consecutive running numbers providing anonymous data replaced the patients' social security numbers. We excluded patients if information on gender was missing (*n* = 47) and if they only received drug formulations not dispensed as multidose dispensed drugs (i.e., inhalers, ointments, mixtures, suppositories, and injectables; *n* = 217), medications exclusively prescribed “as required,” herbal remedies, and medications with unclear dosage (*n* = 34). All drugs were coded according to WHO's Anatomical Therapeutic Chemical (ATC) classification system [14].

### 2.2. Use of Antiepileptic Drugs

We compared the use of AEDs on the substance level between home care service and nursing homes patients by means of percentages of AEDs users and whether patients differed with respect to the use of older versus newer AEDs. AEDs approved before 1990 are regarded as the* older drugs*, namely, carbamazepine, ethosuximide, phenobarbital, phenytoin, primidone, and valproic acid and the benzodiazepines clobazam, clonazepam, and diazepam, whereas all drugs approved after 1990 are defined as the* newer drugs* [15]. We defined patients using one or more AEDs as AED users.

### 2.3. Drug-Drug Interactions Involving AEDs and CNS-Active Drugs

CNS-active drugs included opioid analgesics (N02A), antipsychotics (N05A), anxiolytics (N05B), hypnotics and sedatives (N05C), and antidepressants (N06A). Patients using one or more CNS-active drugs were defined as CNS-active drug users. Concomitant prescribing of CNS-active drugs with AEDs was analyzed at the patient level. We also consulted Norwegian and Danish drug interaction databases for clinical effects and potential consequences of combining AED with other CNS-active drugs. The Norwegian drug interaction database (DRUID) classifies DDIs according to a three-point severity scale: A, should not be combined, B, take precautions, and C, of academic interest [16]. According to this classification, we screened the 882 AED users' drug lists for frequencies of DDIs involving AEDs. In addition, we searched the Danish interaction database for clinical effects of DDIs [17].

### 2.4. Ethics and Approvals

The Norwegian Social Science Data Services approved data collection from the supplier given that they were anonymized. Therefore, the Regional Committee for Medical and Health Research Ethics presented no objections regarding the study design and concluded that committee clearance was not required.

### 2.5. Statistical Analysis

Student's *t*-test was applied to compare means (continuous data: age and number of drugs used) and *χ* ^2^ test was used to compare proportions (categorical data: gender, type of care (home care services versus nursing homes), and older versus newer AEDs). Logistic regression was performed to examine the impact of different factors on the likelihood that older patients in home care service and nursing home were using AEDs (dependent categorical variable: nonuser of AED = 0; user of AED = 1). The model included four independent variables (gender, age, total number of drugs, and setting). We report effect estimates of the impact of settings as prevalence odds ratio (OR) with 95% confidence interval (CI) and *p* < 0.05 significant level. Analyses were performed using SPSS Version 21 (SPSS, Inc., Chicago, IL, USA).

## 3. Results

### 3.1. Population Characteristics

The study population comprised 11,254 patients ≥65 years: 8,268 in home care service (69% women) and 2,986 in nursing homes (72% women). On average, patients in home care service were younger than those in nursing homes (83.0 versus 85.3 years; *p* < 0.001). Women were generally older than men in both home care services (83.8 versus 81.1; *p* < 0.001) and nursing homes (86.3 versus 82.9; *p* < 0.001). Information regarding 63,936 drugs was available, and patients in both settings received a mean of 5.7 regularly dispensed drugs. In the total study population, women received 5.7 drugs and men received 5.5 drugs.

### 3.2. Use of Antiepileptic Drugs

In total, 882 patients (7.8%) used altogether 974 AEDs, with no significant difference between home care services and nursing homes (8.2% versus 7.7%; *p* = 0.383). Of the 882 patients, 801 (90.8%) were monotherapy users, 70 patients (7.9%) used two AEDs, and 11 patients (1.2%) used three AEDs. We identified 13 different AEDs in use, where carbamazepine, pregabalin, lamotrigine, and gabapentin were the most commonly used, accounting for more than 60% of the total AED consumption (see Table 2). Pregabalin and gabapentin were used by 278 (31.5%) of 882 patients. Two AEDs differed in use between settings; gabapentin was more frequently prescribed in home care services (OR: 1.72; CI: 1.09–2.70), while oxcarbazepine was more commonly prescribed in nursing homes (OR: 0.36; CI: 0.15–0.86). When comparing older versus newer AEDs, we observed no significant differences between home care services and nursing homes (older AEDs: 50.5% versus 54.3%; *p* = 0.320). However, in the combined population, men used older AEDs more frequently than women (56.6% versus 48.4%; *p* = 0.018); patients taking AEDs were younger than nonusers (79.0 versus 84.0 years; *p* < 0.001) and also used more drugs (7.0 versus 5.6 drugs; *p* < 0.001, Table 1).

### 3.3. Drug-Drug Interactions Involving AEDs and CNS-Active Drugs

In the combined population, 436 different DDIs were present in 45% of the patients using AED. We identified 79 different pharmacokinetic interactions involving AEDs drugs with other CNS-active drugs (Table 3). Patients using AEDs were more frequently prescribed CNS-active drugs compared to non-AEDs users (9.2% versus 6.0%; *p* < 0.001), and this combination was more often prescribed to men than women (12.2% versus 8.1%; *p* < 0.001). Antidepressants (6.6%), antipsychotics (3.0%), and opioids (2.3%) were all more frequently prescribed among users of AEDs. Seventy-nine patients (9.0%) were prescribed AEDs concomitantly with opioids (N02A).

### 3.4. Antiepileptic Drugs Involved in Other Drug-Drug Interactions

We identified 357 other potential DDIs involving AEDs. Of these, also here 79 were pharmacokinetic interactions, 265 were pharmacodynamics interactions, and 13 were classified as other (Table 4). Four of the identified AEDs, carbamazepine, phenobarbital, phenytoin, and valproic acid, are habitually involved in pharmacokinetic DDIs (*n* = 151), while pregabalin and gabapentin are more prone to pharmacodynamics interactions (*n* = 206). Simvastatin (*n* = 35) and zopiclone (*n* = 109) were the drugs most prevalently involved in pharmacokinetic and dynamic interactions, respectively.

## 4. Discussion

In this large study of home care service and nursing home patients, we identified similar use of AEDs among patients in both settings but higher use as compared to the general older Norwegian population [13]. The use of older versus newer AEDs did not vary significantly between home care services and nursing homes. The most commonly used AED was carbamazepine, which is defined among the older drugs. Newer AEDs, however, should in many cases be preferred, as they have a lower potential for causing enzyme induction and inhibition; they usually have more simple pharmacokinetic properties and rarely cause serious adverse effects [6]. In nearly half of all patients, AEDs were involved in potential DDIs. This highlights the importance of awareness of DDIs to identify and to monitor potential harmful effects in the individual patient among the elderly.

### 4.1. Total Drug Consumption

The average use of 5.7 multidose dispensed drugs in elderly patients in home care services and nursing homes reflects similar comorbidity between the two settings. The association between polypharmacy (≥5 drugs), use of inappropriate drugs, and adverse drug reactions is well known and calls for attention when changing drug therapy in frail elderly patients [18]. Based on medication reviews in nursing homes, common drug-related problems include unnecessary drug use, dose adjustments (especially dose reductions), need for monitoring, and unclear drug charts [19]. For elderly patients who are prescribed AEDs, there is an increased risk to experience DDIs and drug-disease interactions, and particular attention should be given to both [9, 20].

### 4.2. The Use of Antiepileptic Drugs

This study demonstrates that patients in home care services or residing in nursing homes use AEDs frequently for indications other than epilepsy. In total 7.8% of the patients in both settings were prescribed AEDs, which is more than twice as prevalent (3.4%) as compared to general older Norwegian population ≥65 years [13]. The use of AEDs during the last ten years also reflects prescribing patterns in other European countries. Other studies of elderly patients in, for example, German and US nursing homes, report AED use to vary between 5 and 11% [21, 22], while community-dwelling elderly and elderly patients in Swedish institutions use 2% and 9%, respectively [23]. Both the incidence and prevalence, 9.0/1000 in a study including patients with 18–82 years [24] versus 6.6/1000 in a study of patients from 0 to 12 years [25], of epilepsy are higher but stable in older patients compared to younger patients [26–28].

There were no differences in the use of AEDs between patients in home care services and nursing homes, except for two drugs: gabapentin, used more frequently in home care service patients, and oxcarbazepine, used more frequently in nursing home patients. Treatment with gabapentin probably reflects use in neuropathic pain, while oxcarbazepine is primarily indicated in focal epilepsies [29]. According to recent updated treatment guidelines, gabapentin and lamotrigine have a* level A* evidence for the treatment of focal epilepsies in the elderly [30], while there are no studies at present for oxcarbazepine. It seems reasonable that the frequent use of oxcarbazepine is an alternative to the most commonly used AED, carbamazepine, since oxcarbazepine has no autoinduction or strong enzyme-inducing properties [9]. Furthermore, the use of older compared to newer AEDs was similar and did not vary significantly between home care services and nursing homes. Other studies have reported that older AEDs are more frequently used in the elderly [29], while pregabalin and gabapentin, both newer agents, are commonly used in neuropathic pain [31].

Patients who were prescribed AEDs compared to non-AED users were using CNS-active drugs more frequently, in line with studies of higher prevalence of psychiatric comorbid disorders in patients with epilepsy [29, 32]. The high percentage of coprescribing of psychotropic drugs with lamotrigine points to an extensive use in psychiatric indications and opioid analgesics with gabapentin or pregabalin in treatment of pain, both consistent with earlier observations in the Norwegian population [2]. A large Norwegian study including more than 445.000 elderly patients reported that 35% received potentially inappropriate medications. As more than half of these inappropriate medications were psychoactive substances [33], elderly AED users may have an increased risk to experience drug-related problems.

### 4.3. Antiepileptic Drugs Involved in Other Drug-Drug Interactions

Careful considerations and profound pharmacological knowledge are necessary when combining AEDs or whenever changing AED therapy. The prevalence of DDIs in our study was 45% within patients using an AED. Most of the AEDs (i.e., carbamazepine, phenobarbital, and phenytoin) involved in DDIs induce the metabolism of other drugs, resulting in decreased serum concentrations of a large number of drugs [34, 35]. For instance, zopiclone in combination with phenobarbital or phenytoin may, due to pharmacokinetic properties, lead to a reduction in total serum concentration of zopiclone. Contrarily, zopiclone combined with carbamazepine increases sedation, as well as other CNS-depressant effects, that is, causing a pharmacodynamic interaction.

### 4.4. Clinical Considerations

This large study of elderly patients in need of health services obtained detailed information of drug use based on a multidose dispensed drug registry. Comprehensive datasets comprising vulnerable older patients are difficult to retrieve and are seldom easily available. For instance, the Norwegian Prescription Registry does not include patients from nursing homes. Thus, these data can be especially useful for further clinical assessment of importance for prescribers and clinical pharmacists considering rational drug therapy in this patient group. Our study shows that, quantitatively, the potentials for pharmacokinetic and pharmacodynamics interactions are highly common in elderly patients receiving AEDs. DDIs in general may affect the fine-tuned balance between efficacy and tolerability. Pharmacokinetic interactions lead to alterations in the serum concentrations of various other drugs as summarized, but the individual response and the clinical significance are difficult to predict. Additionally, pharmacological effects or adverse effects due to pharmacodynamic interactions make it even more challenging to predict a treatment outcome in the individual patient. The combination of several CNS-active drugs, as well as the possibility of excessive adverse drug reactions, for example, sedation and impaired cognitive function, advocates close monitoring. Studies on medication review in elderly recommend dose adjustments or reducing the number of CNS-active drugs given concomitantly [19].

Our findings highlight the importance of awareness of excessive adverse effects or toxicity when giving AEDs in polytherapy. The increase in total drug load calls for attention towards pharmacodynamic and pharmacokinetic drug interactions, which subsequently may lead to alterations of effects and adverse drug reactions. Conversely, ending drug treatment could also lead to adverse drug reactions as a result of ongoing DDI processes [1]. Therefore, clinicians should be aware of drug combinations with susceptibility of serious clinical outcomes. For instance, it is important with strategies to avoid combinations including carbamazepine, phenobarbital, or phenytoin, particularly since these drugs often cause therapeutic failure with other drugs if they were not dosage-adjusted. It is also important to systematically reconsider and decrease the dosage of all CNS-active drugs to minimize adverse effects and take into account pharmacokinetic changes, that is, absorption, distribution, and elimination alterations, with increasing age, in particular, since elderly patients demonstrate even more extensive variability in pharmacokinetics than younger adults [9, 36]. AEDs that are often used in this patient group, and as the present results prove, include gabapentin and pregabalin, where extensive variability in serum concentration versus dose ratios recently was shown [37]. Based on the present results we recommend that all elderly people receiving care services should have the possibility of having their medications reviewed by pharmacists at regular intervals to limit the number of drugs and ensure that DDIs are prevented. Furthermore, there should be a concerted effort to replace older AEDs with newer AEDs in this population to avoid the risk of interactions caused by enzyme-inducing AEDs (carbamazepine, phenytoin, and phenobarbital). Finally, therapeutic drug monitoring should be included as a standard of care for all elderly patients receiving AEDs.

### 4.5. Strengths and Limitations

A major strength of this study is the large number of patients included representing approximately 30% of all patients receiving multidose dispensed drugs in Norway in 2009. Since there are no registries where these elderly patients are included, detailed studies regarding their use of drugs in real life are often difficult to conduct. The representativeness could have been improved by including patients from the other two multidose dispensed drug suppliers. However, we do not have any indications that the selected supplier differs with regard to patient population. Prescribing patterns might differ when it comes to multidose dispensed drugs versus ordinary drug dispensing, as the system does not seem to reduce the number of errors in transferring data about medication between health care levels [38].

This large study population gives the opportunity to gain information on drug utilization in a new way, and we could quantify the actual number of AEDs and other CNS-active drugs in use and point to the risk of interactions between these drugs. The large number of possible interactions that may occur and the fact that this is a risk factor in 45% of the patients point to a need for careful clinical considerations in the individual patient and observations with focus on adverse effects by health care professionals.

We included 10% of the total elderly population receiving home care services and nursing homes, thus reflecting AED use in older persons in these two care settings. However, comparison with other studies is hampered as we used multidose dispensed drug data. For instance, information regarding “as required” medications and other formulations than capsules and tablets was not available (e.g., not including rectal administration of diazepam) and thus was not included in the analyses. Although the data were collected retrospectively, there are no indications that large changes in prescriptions of AEDs in the elderly have occurred since data collection. Another limitation concerns the lack of diagnostic and clinical data at the individual level. However, regardless of indication, the risk of DDIs is of importance for most AEDs and is often considered clinically relevant.

## 5. Conclusions

In a large population of elderly, home care service and nursing home patients do not differ with respect to exposure of AEDs, but they use more AEDs as compared to the general population of similar age. The risk of DDIs with AEDs and other CNS-active drugs should be taken into consideration and individual clinical evaluations were assessed in this population. The use of older versus newer AEDs was similar and did not vary significantly between the two settings. The fact that 45% of the patients had potential DDIs involving AEDs and other CNS-active drugs stresses the need for cautiousness when initiating new therapy and for regular medication reviews to assess the need for dose adjustments or deprescribing in elderly. In total the potentials for 436 pharmacokinetic/dynamic AED interactions were identified. These findings call for close monitoring in the individual patient to avoid excessive adverse effects or toxicity reactions. This is an important concern for prescribers and clinical pharmacists when considering rational therapy in the elderly.

## Figures and Tables

**Table 1:** Patient characteristics, total use of multidose dispensed drugs, and distribution of antiepileptic drugs by gender among elderly patients ≥65 years in nursing homes and home care services (*n* = 11254).

	Total group		Nursing homes						Home care services					
			Total		Men		Women		Total		Men		Women	
*Population*														
≥65 years	11254	100	2986	100	843	28.2	2143	71.8	8268	100	2566	31.0	5702	69.0

*Non-AED users*														
*n*, %	10372	92.1	2741	91.8	753	89.3	1988	92.8	7631	92.3	2315	90.2	5316	93.2
Age (M), SD	84.0	7.2	85.7	7.1	83.4	7.3	86.6	6.8	83.4	7.1	81.6	7.4	84.1	6.8
Drugs (M), SD	5.6	2.5	5.6	2.7	5.6	5.6	5.6	2.7	5.6	2.4	5.4	2.4	5.6	2.5

*AED users*														
*n*, %	882	7.8	245	8.2	90	10.7	155	7.2	637	7.7	251	9.8	386	6.8
Age (M), SD	79.0	8.2	81.0	8.3	78.9	7.1	82.3	8.8	78.2	8.1	76.4	7.9	79.4	8.0
Drugs (M), SD	7.0	2.8	7.3	3.0	7.1	2.8	7.3	3.1	7.0	2.7	6.6	2.7	7.2	2.6

AED, antiepileptic drug; M, mean; SD, standard deviation.

**Table 2:** Antiepileptic drug use among patients (*n* = 882) in home care services (HCS) and nursing homes. The association (odds ratio, OR) and 95% confidence interval (CI) for use of the drugs, adjusted for patients' age, gender, and total number of drugs used in different settings (home care service used as reference).

Drug	Home care services (*n* = 637)		Nursing homes (*n* = 245)		All patients (*n* = 882)		OR	CI
	*n*	%	*n*	%	*n*	%		
Carbamazepine	134	21.0	55	22.4	189	21.4	0.88	0.61–1.26
Pregabalin	95	14.9	50	20.4	145	16.4	0.80	0.54–1.18
Lamotrigine	102	16.0	32	13.1	134	15.2	1.15	0.74–1.79
Gabapentin	104	16.3	29	11.8	133	15.1	1.72	1.09–2.70
Valproate	66	10.4	27	11.0	93	10.5	0.78	0.48–1.26
Clonazepam	60	9.4	27	11.0	87	9.9	0.79	0.49–1.29
Phenobarbital	48	7.5	15	6.1	63	7.1	1.26	0.68–2.31
Levetiracetam	49	7.7	12	4.9	61	6.9	1.46	0.76–2.81
Phenytoin	29	4.6	10	4.1	39	4.4	1.08	0.51–3.99
Oxcarbazepine	11	1.7	10	4.1	21	2.4	0.36	0.15–0.86
Topiramate	4	0.6	1	0.4	5	0.6	1.29	0.14–11.83
Zonisamide	3	0.5	0	0	3	0.3	—	—
Primidone^*∗*^	1	0.2	0	0	1	0.1	—	—

*Total*	*706*		*268*		*974* ^**∗∗**^			

^*∗*^Not marketed in Norwegian. ^*∗∗*^Some patients used more than one antiepileptic drug. OR, odds ratio; CI, confidence interval (95%).

**Table 3:** Mechanisms, clinical consequences, severity, and frequencies of pharmacokinetic drug-drug interactions involving antiepileptic drugs and other CNS-active drugs identified in patients ≥65 years (*n* = 882) in home care services and nursing homes.

Antiepileptic drug	Substrates for these enzymes and possible interactions	Clinical effects and potential consequences^#^	Severity^$^	Frequencies
*Enzyme inducers*				

*Carbamazepine, n = 189*				
Enzyme-inducing properties, CYP 3A4, 2C9, 1A2, or UGTs	Benzodiazepines	Decrease in serum concentrations of substrates of these enzymes and possibly a decrease in clinical efficacy.	B	32
	Haloperidol	Dose adjustment needed. Decreased serum concentration of haloperidol by 50% but varies with dose of carbamazepine.^$^	B	7
	Lamotrigine	Dose adjustment needed. Decreased serum concentration of lamotrigine by 50%.	B	6
	Amitriptyline	Dose adjustment needed. Decreased serum concentration of amitriptyline by 50–60%.^$^	B	5
	Risperidone	Dose adjustment needed. Serum concentration of risperidone and active metabolite reduced by 65% and 50%, respectively.	B	4
	Quetiapine	Dose adjustment needed. Maximum serum concentrations of quetiapine reduced by nearly 80%.	A	3
	Valproic acid	Dose adjustments needed for both drugs. Carbamazepine concentration increased by 25–50%, while valproate concentration reduced by 30%.	B	2

*Phenobarbital, n = 63*				
Enzyme-inducing properties, CYP 3A4 or UGT	Haloperidol	Dose adjustment needed because of decreased serum concentration of haloperidol by 50%.	B	2
Enzyme inhibitory properties on CYP 2C19^*∗*^	Risperidone	Dose adjustment needed. Increased serum concentration of risperidone and thus a potential increase of adverse effects or toxicity.	B	1

*Phenytoin, n = 39*				
Enzyme-inducing properties, CYP 3A4 or UGT	Haloperidol	Dose adjustment needed. Decrease in serum concentrations of substrates of these enzymes and possibly a decrease in clinical efficacy because of decreased serum concentration of haloperidol by 50%.	B	1

*Enzyme inhibitors*				

*Valproic acid, n = 93*				
Enzyme inhibitory properties on CYP 2C9/19, 3A4?, UGTs	Lamotrigine	Dose adjustment needed because of an increase in the serum concentrations of substrates of these enzymes. Clearance of lamotrigine reduced by 50%, potentially causing skin rashes and neurotoxic effects.	B	11
	Amitriptyline, carbamazepine	Dose adjustment needed because of decrease of first-pass metabolism of amitriptyline. For carbamazepine, see above.	B	2^**∗∗**^
	Clomipramine, phenobarbital, phenytoin	Dose adjustment needed because clearance of phenobarbital reduces by 40% and thus there is a risk of intoxication.	B	3^**∗∗**^

*In total*				*79* ^*∗∗*^

Clinical effects and potential consequences based on Norwegian and Danish interaction databases in addition to a review by Johannessen and Landmark, 2010 [1]. ^*∗*^Weak inhibition based on Johannessen and Landmark 2010 [1]. ^#^According to the Danish drug interaction database. ^$^The Norwegian interaction database (DRUID) denotes severity of drug-drug interactions according to a three-point severity scale: A, should not be combined, B, take precautions, and C, of academic interest. Herein, only drug-drug interactions in categories A and B are shown. Drug-drug interactions involving the antiepileptic drugs felbamate (with carbamazepine, diazepam, phenobarbital, phenytoin, and valproic acid), oxcarbazepine (with lamotrigine, phenobarbital, and phenytoin), rufinamide (with carbamazepine and felbamate), stiripentol (with carbamazepine, felbamate, phenobarbital, phenytoin, and valproic acid), topiramate (>200 mg/day with phenytoin), and valproic acid (with clozapine, imipramine, nortriptyline, and rufinamide) were not identified and therefore not included. ^**∗∗**^Duplicates not shown and not counted.

**Table 4:** Other identified drug-drug interactions involving antiepileptic drugs in patients ≥65 years receiving home cares services or living in nursing homes.

	Antiepileptic drugs										
	Carbamazepine		Phenobarbital		Phenytoin		Valproic acid		Pregabalin or gabapentin		Total
	*n* = 189		*n* = 63		*n* = 39		*n* = 93		*n* = 278		
	*n*	%	*n*	%	*n*	%	*n*	%	*n*	%	
*Pharmacokinetic interactions* ^1^											**79**
Amlodipine	19	10.0	4	6.3							23
Atorvastatin			3	4.8							3
Simvastatin	31	16.4			4	10.3					35
Folic acid					5	12.8					5
Zopiclone			9	14.3	4	10.3					13

*Pharmacodynamic interactions* ^2^											**265**
Benzodiazepines					4^*∗*^	10.3			49	17.6	53
Fluoxetine							1	1.1			1
Mianserin	13	6.9	2	3.2	2	5.1					17
Opioids			6	9.5					79	28.4	85
Zopiclone	31	16.4							78	28.1	109

*Other interactions* ^3^											**13**
AII-blockers and diuretics	13	6.9									13

*Interactions in total*	*107*		*24*		*19*		*1*		*206*		*357*

^1^Caused by combination with AED enzyme inducers or inhibitors causing a decrease in serum concentration and lack of efficacy of the affected drug or an increase in serum concentration and excessive adverse effects or toxicity, respectively. ^2^With other CNS-active drugs resulting in a possibility for excessive adverse effects and sedation. ^3^Increased excretion of sodium and increased risk of hyponatremia with carbamazepine. ^*∗*^Also a pharmacokinetic interaction and included in Table 3.

## Abbreviations

AED: Antiepileptic drug
ATC: Anatomical Therapeutic Chemical classification system
CNS: Central nervous system
DDI: Drug-drug interactions
NorPD: Norwegian prescription database
OR: Odds ratio.
